# Motion and Interaction of Magnetic Dislocations in Alternating Magnetic Field

**DOI:** 10.1038/s41598-017-18033-2

**Published:** 2017-12-22

**Authors:** L. A. Pamyatnykh, B. N. Filippov, L. Y. Agafonov, M. S. Lysov

**Affiliations:** 1 Ural Federal University, Lenin Av. 51, Ekaterinburg, 620083 Russia; 2 Mikheev Institute of Metal Physics, Ural Branch, Russian Academy of Sciences, Ekaterinburg, 620219 Russia

## Abstract

The behavior of magnetic dislocations (MDs) in an alternating harmonic magnetic field in iron garnets has been experimentally investigated. The results are presented for single-crystal plates in which the drift of domain walls is observed in fields of sound frequencies. It is found that MDs in a stripe domain structure are able to move not only along but also across domain walls. A pairwise interaction between magnetic dislocations when they approach each other to distances on the order of the sizes of the cores of MDs is revealed. The processes of the annihilation, mutual passing of magnetic dislocations through each other and overtaking are found. The features of the dynamic behavior of MDs are explained using a mechanism based on the presence of vertical Bloch lines in a structure of domain walls. MDs are formed at nucleation centers, and their nucleation field is lower than the drift-starting field, which corresponds to previously proposed dislocational mechanism of the drift. The dependencies of quantitative parameters of the drift and MDs on amplitude and frequency of the pumping field are determined. The behavior of MDs should be considered when analyzing the mechanisms for magnetization and temperature-dependent phase transitions in magnetic layers.

## Introduction

A domain structure (DS) exerts a significant effect on many important characteristics of magnetically ordered materials^1–8^. As shown in^9,10^, a defect of the domain structure–magnetic dislocations (MDs)–defines the mechanism for the drift of domain walls (DWs) in an alternating magnetic field. In this work, we report a study of the dynamic behavior of magnetic dislocations themselves. A magnetic dislocation is a specific analogue of crystal lattice linear defects called edge dislocations. The latter occur as the result of the partial removal of one of the parallel atomic planes^11^.

One of the first papers in which magnetic dislocations were considered is^12^. However, the analysis reported in^12^ was performed for isotropic media; therefore, it has limited applicability to real magnetics. A successive theoretical consideration of the formation, annihilation, and movement of magnetic dislocations in static external magnetic fields and an experimental study of these processes in epitaxial iron garnet films subjected to constant-valued magnetic fields can be found in^13,14^.

The role of MDs in changing the type of a DS under varying simulation temperatures was theoretically studied in^15,16^. In those works, MDs were viewed as topological defects, and a change in the DS ordering type from stripes to labyrinth was considered to be a Kosterlitz-Thouless topological phase transition. A calculation of the distortions of the shape of DWs that appear in a stripe-type DS when an MD is present was performed in^16^ in the framework of the Ising model with a dipole interaction. It was shown that two MDs can interact with each other by virtue of these distortions, even when separated by a distance. The mechanisms for the transformations of a static DS that are observed when the temperature is varied were considered in^17,18^.

There are currently no papers devoted to studying the dynamic behavior of MDs in alternating magnetic fields. Our proposed study is the first to experimentally consider this problem. Investigations were performed on anisotropic single-crystal plates of rare-earth iron garnets subjected to alternating magnetic fields. The results presented herein were obtained on a quasiuniaxial (111) plate that was cut from a (TbErGd)_3_(FeAl)_5_O_12_ single crystal. The domain structures were visualized with the magneto-optical Faraday effect and were recorded using a high-speed camera. The motion of MDs in an alternating magnetic field of sound frequencies (*f* = 25 − 1200 Hz) was tracked.

New effects related to the motion of MDs across a stripe-type DS are reported. A new mechanism for the change in the magnetization distribution during the dynamic magnetization and remagnetization of a crystal is described. This mechanism is the movement of an MD in a system of stripe domains accompanied by the switching of the magnetization of an MD. For the quantitative description of this process, the following characteristics of the dynamic motion of the domain walls were measured: velocity of the drift of domain walls and speeds of the motion of MDs upon variations in the frequency and amplitude of the magnetic field. The process of nucleation of MDs in an alternating magnetic field was also studied.

## Results

First, analogous to the case of edge dislocations in a crystal lattice, we introduce the concepts of the MD core and the MD Burgers vector. We define the MD core as an area associated with the end of a shortened domain. Inside this area, the domain walls are curved, and their energy differs from that of straight DWs. The MD Burgers vector $\overrightarrow{b}$ is used in a manner analogous to^12^ (see the Methods section for details).

In the range of comparatively low frequencies (25–1200 Hz) of an alternating magnetic field, we observe drift of DWs, i.e., the system of stripe domains moves as a whole in a direction perpendicular to the planes of DWs^9,10^. In this work we study not the drift of DWs itself but nucleation, motion and interaction of MDs.

**Figure 1:**
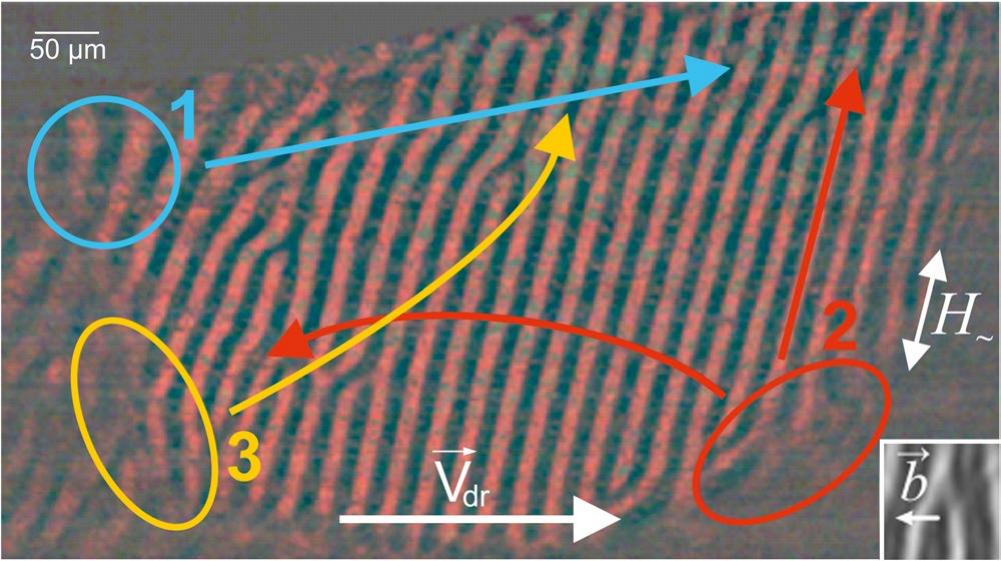
Centers of nucleation of magnetic dislocations. Areas where magnetic dislocations form are encircled. The most frequently observed trajectories of movement for magnetic dislocations nucleated in corresponding centers are shown with color arrows. White arrow indicates the direction of the drift of domain walls. The inset presents an example of a magnetic dislocation and its Burgers vector.

In the studied range of frequencies, MDs nucleated in the three centers located as shown in Fig. 1. Direct observations indicated that local sample areas where MDs nucleate have transverse dimensions (length and width) that are on the order of units of stripe DS periods. The arrows in Fig. 1 indicate the most frequently observed trajectories of movement of MDs. The inset shows a Burgers vector designation for an MD. Magnetic dislocations move through a stripe DS both parallel and perpendicular to DWs, and in most cases, they have velocity components in both specified directions.

Observations have revealed the following:

1. The speed of movement of MDs can exceed the speed of drift of DWs by an order of magnitude. Both speeds are defined by the amplitude and frequency of the field.
2. The activity of the centers *I*_*MD*_ (the number of MDs nucleated in a certain nucleation center during one second) depends on *H*_0_ and *f*. The maximum total number of MDs nucleated in all nucleation centers was measured at 900 Hz and equals *I*_*MDS*_ = 316 MDs per second.
3. The interaction of MDs with each other and with the stripe domains of an initial DS is found. Only the following simplest scenarios of the behavior of MDs will be considered in this paper:

- A magnetic dislocation moves through a system of stripe domains, and two magnetic dislocations pass through each other while moving in opposite directions;
- Two MDs moving toward each other annihilate;
- One magnetic dislocation overtakes the other while they move in the same direction.

Next, we will consider these simplest scenarios of the behavior of MDs in detail.

### Movement of a single magnetic dislocation through a system of stripe domains

**Figure 2:**
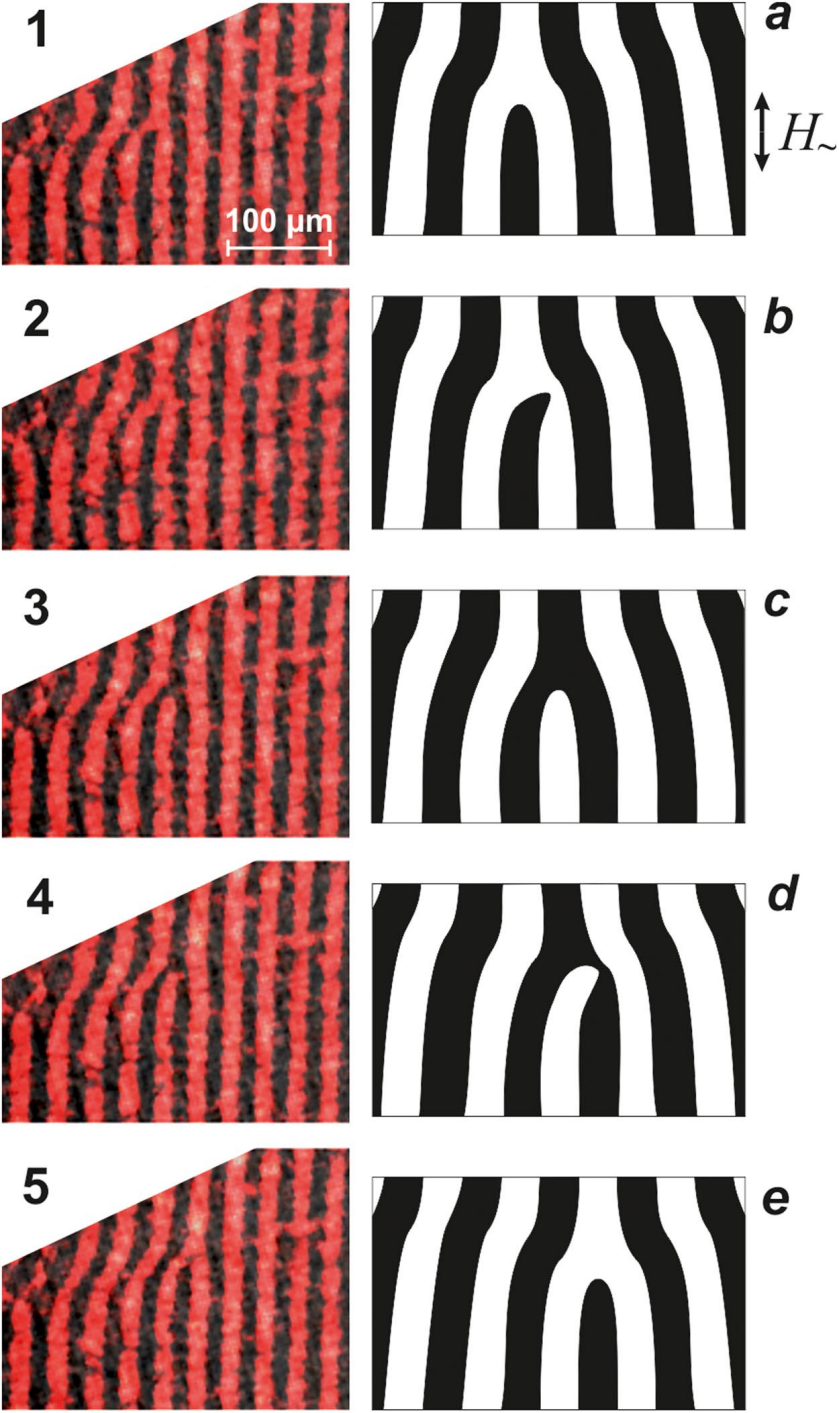
Single magnetic dislocation moving through a system of stripe domains. The process occurs in an alternating magnetic field oriented along domain walls in the sample plane. The AC field amplitude is *H*_0_ = 80 Oe, and the frequency is *f* = 200 Hz. The time between frames equals (**1**-**2**) 2, (**2**-**3**) 28, (**3**-**4**) 5, and (**4**-**5**) 5 ms.

The observed movement of a single MD that occurs under the influence of an alternating magnetic field directed along the domain walls is shown in Fig. 2. The experimental images (Fig. 2(1–5)) and their schematic interpretations (Fig. 2(a–e)) are provided. In the initial state, there is a “dark” dislocation (Fig. 2(1,a)) that is magnetized “into the plane of the figure”. Then, the free end of the shortened “dark” domain stretches by forming a narrow bridge (Fig. 2(2,b)) and connects to a dark stripe domain on the right, thereby forming a “bright” magnetic dislocation (Fig. 2(3,c)) that is magnetized “outward from the plane of the figure”. Next, the free end of the shortened “bright” domain connects to a bright stripe domain on the right. The latter occurs through stretching of the free end of the bright short domain (Fig. 2(4,d)). Thus, a single dark dislocation turns out to have been shifted by one period of a stripe DS (cf. Fig. 2(1,a) and Fig. 2(5,e)). The process described above repeats, and the MD moves from the left to the right.

Thus, the observations have revealed that a single magnetic dislocation in an alternating magnetic field moves across a stripe DS, successively interacting with all magnetic domains that it encounters along the way. One very important feature of this interaction is the periodic reorientation of the MD magnetization vector.

### The processes of interaction of magnetic dislocations

Apart from an interaction of MDs with stripe domains of the initial DS, their (MDs) interaction with each other was also found. Let us consider the observed scenarios of an interaction of MDs.

**Figure 3:**
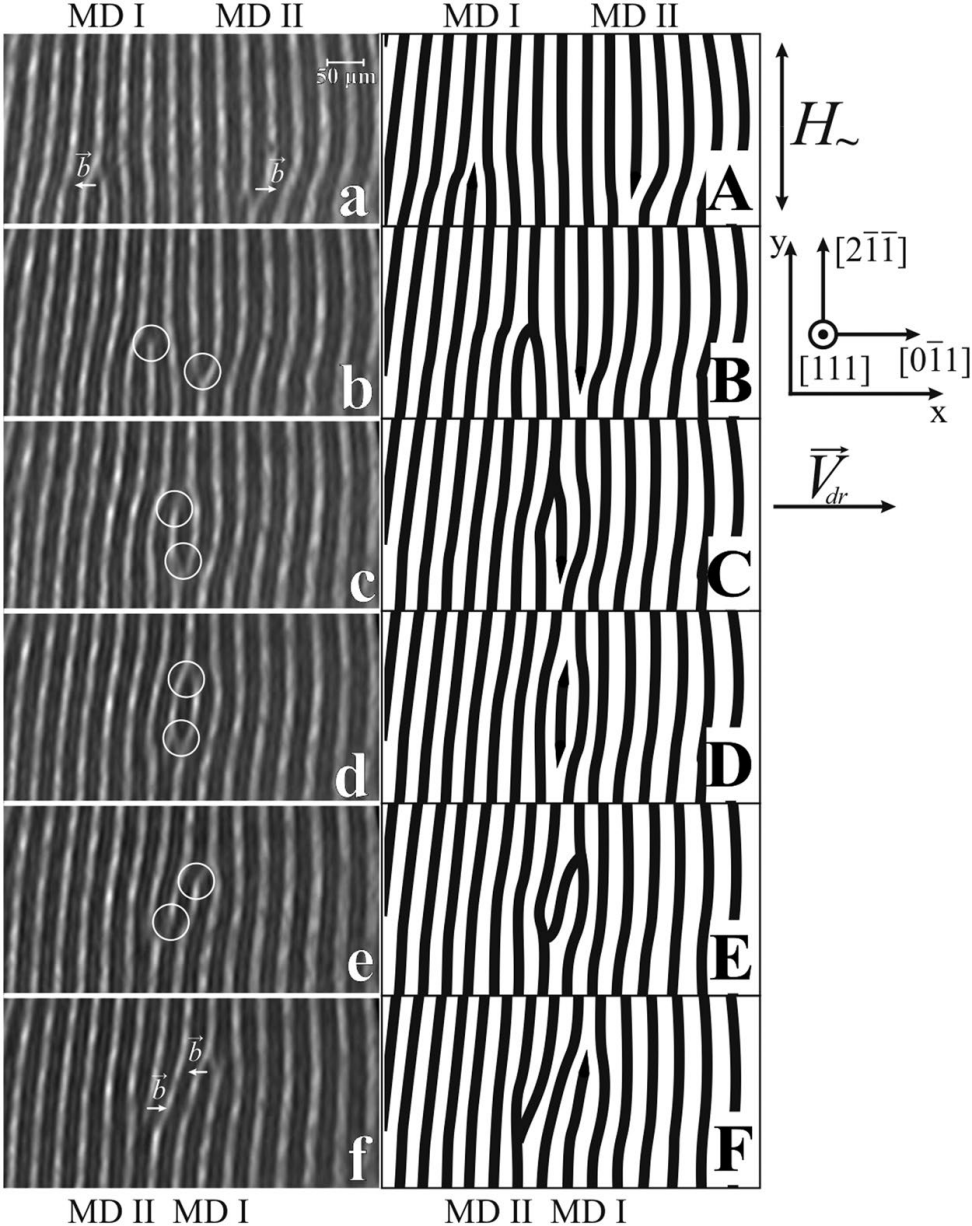
Two magnetic dislocations moving toward each other. The Burgers vectors $\overrightarrow{b}$ of magnetic dislocations are aligned in opposite directions. (**a**–**f**) Experimental magneto-optical images of an iron garnet sample domain structure in an AC magnetic field. The AC field frequency is *f* = 60 Hz, and the amplitude is *H*_0_ = 106 Oe. The Burgers vectors of magnetic dislocations are shown in (**a**,**f**). The cores of magnetic dislocations are encircled in (**b**–**e**). The time between frames equals (**a**-**b**) 40, (**b**-**c**) 20, (**c**-**d**, **d**-**e**, and **e**-**f**) 10 ms.

**Figure 4:**
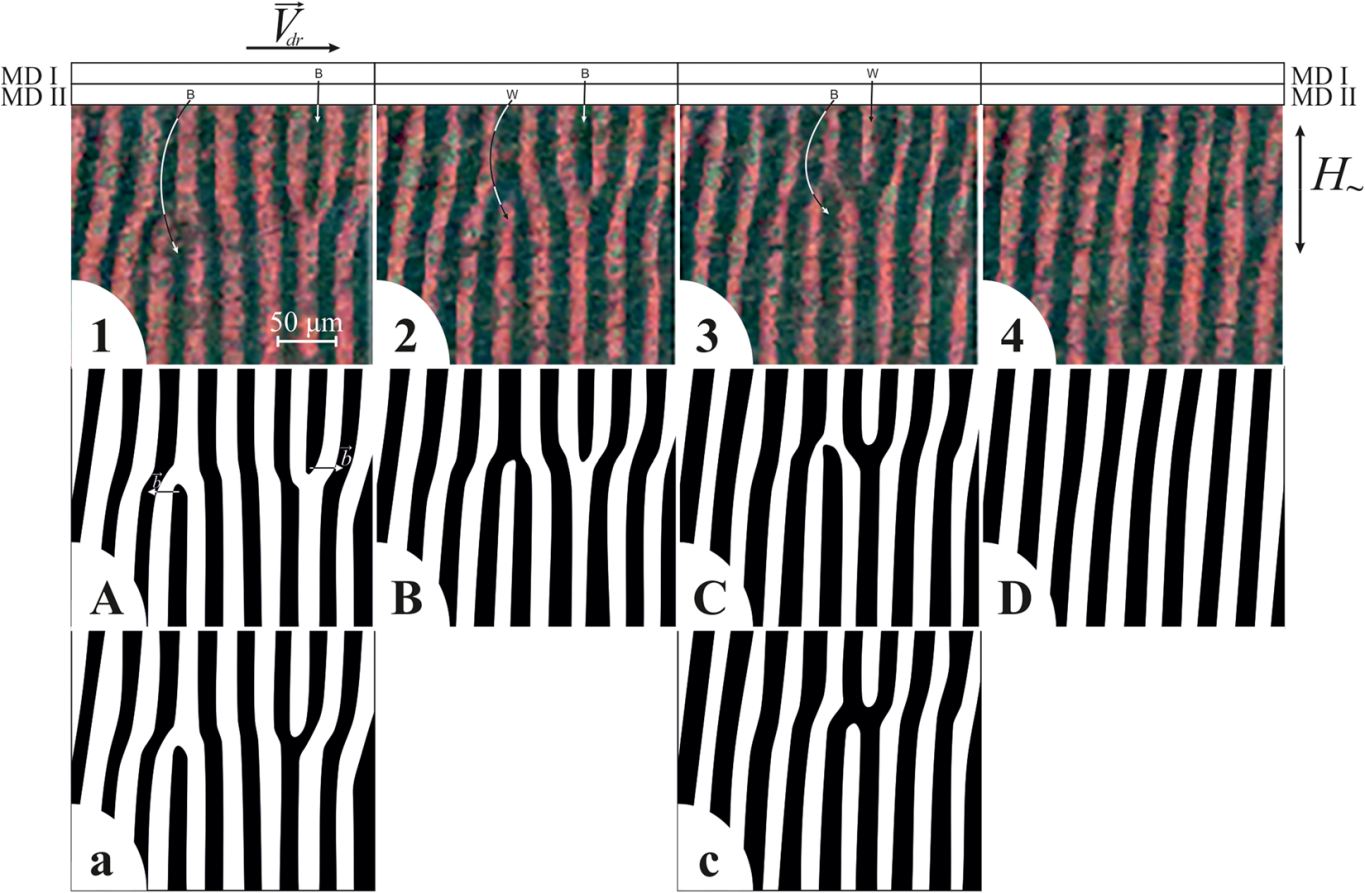
Two magnetic dislocations moving toward each other and their annihilation. The AC field frequency is *f* = 200 Hz. (**1**–**4**) Experimental magneto-optical images of domain structure. The positions of magnetic dislocations MD I and MD II along the *X* axis and their type (Black/White) are indicated in the top part of the figure. The time between frames equals 10 ms. (**A**,**B**,**C**,**D**) Schematic representation of corresponding domain structure states. (**a**,**c**) Supposed intermediate states between frames (**1**,**2**) and (**3**,**4**) based on observations, correspondingly.

**Figure 5:**
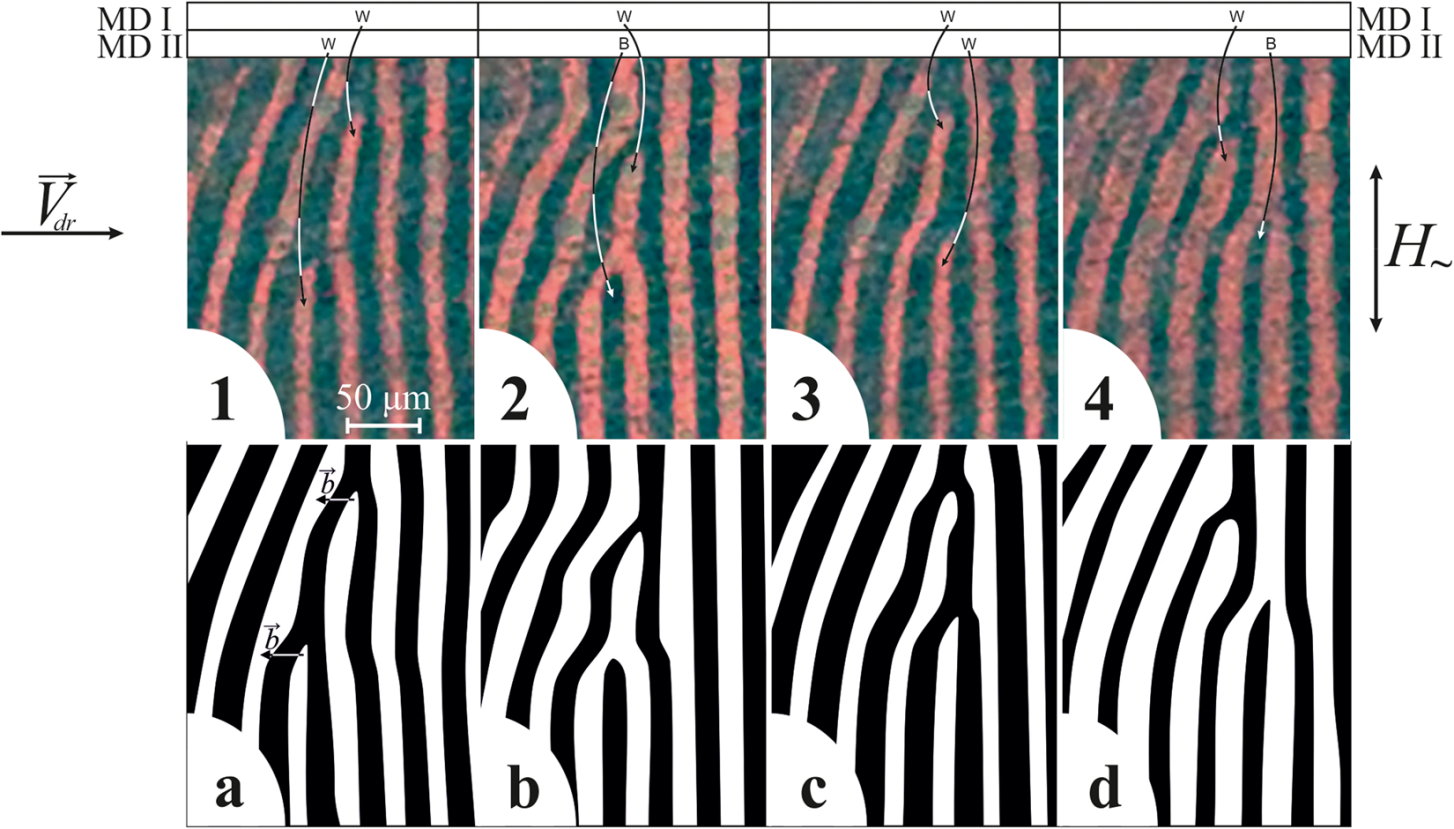
One magnetic dislocation overtakes the other. (**1**–**4**) Experimental magneto-optical images of domain structure. (**a**–**d**) Schematic representation of corresponding domain structure states. The positions of magnetic dislocations MD I and MD II along the *X* axis and their type (Black/White) are indicated in the top part of the figure. The Burgers vectors of magnetic dislocations are shown in (**a**).

Figures 3 and 4 show scenarios of interactions between MDs that are observed in the case where the Burgers vectors of two MDs are aligned in opposite directions. An example of an interaction of two MDs in the case where their Burgers vectors are aligned in the same direction is shown in Fig. 5.

A set of frames in Fig. 3 illustrates the actually observed consequential movement of two MDs toward each other in an alternating magnetic field (*f* = 60 Hz, *H*_0_ = 106 Oe). A coordinate system (*X*-*Y*) bound to the sample is introduced. The *Y* axis is directed along the $\mathrm{[2}\bar{1}\bar{1}]$ axis that is of an average orientation of DWs. The movement of two dislocations is being tracked (see Fig. 3). The cores of the two MDs are encircled in the experimental frames and are labeled as MD I and MD II near the apexes of the shortened domains in Supplementary Fig. 1S. For the initial *Y* coordinates of the MDs, *Y*_*I*_ > *Y*_*II*_. The same elementary acts of interaction between an MD and the stripe domains that were described above for a single dislocation occur here when the two MDs move toward each other. We will only note the positions of the two MDs under consideration for successive moments of time without repeating the detailed description of these elementary acts of interaction.

Frames a–f show the mutual positions of magnetic dislocations in successive moments of time. The time between adjacent frames is a multiple of *t* = 0.01 seconds. Schemes A–F in Fig. 3 show the configurations of the domains in frames a–f, respectively. Schemes that illustrate the observed transformations of a DS in more detail are presented in Supplementary Fig. 1S. As shown, the distance between dislocations decreases with time as they move toward each other in the following manner.

1. At the beginning (Fig. 3(a)), we distinguish two (“dark”) magnetic dislocations, MD I and MD II, i.e., the dislocations with their magnetization directed along −$\overrightarrow{n}$.
2. After *t* = 0.04 seconds, MD I became “bright”, i.e., its magnetization has reoriented and is now directed along $\overrightarrow{n}$. MD II shifted to the left and remained “dark” (Fig. 3(b)).
3. After *t* = 0.02 seconds, MD I is “bright” and MD II is “dark” (Fig. 3(c)).
4. After *t* = 0.01 seconds, MD I and MD II are both “dark” (Fig. 3(d)). In this state, the *X* coordinates of the two magnetic dislocations are equal, and their magnetization is directed along −$\overrightarrow{n}$.
5. After *t* = 0.01 seconds, as shown in Fig. 3(e), magnetic dislocations “fall apart”. Both MD I and MD II are “bright”.
6. After *t* = 0.01 seconds, the distance between the dislocations increases. As shown in Fig. 3(f), MDI is “dark” and MD II is “bright”. There are two stripe domains (of opposite “colors”) between them.

We interpret this observed movement of MDs as two MDs passing through each other (compare Fig. 3(b) and (f)).

Consider another process in which two MDs with oppositely aligned Burgers vectors move toward each other, but in contrast to the previous case, the initial *Y* coordinates of MD I and MD II are equal (*Y*_*I*_ ≈ *Y*_*II*_). This process and its clearer schematic representation are shown in Fig. 4. As shown in this figure, two initially dark dislocations move toward each other and annihilate. This situation results in the appearance of the regular stripe structure (Fig. 4(D)). Here, the elementary acts of interaction between MDs and the stripe domains are the same as those when the movement of a single MD is considered. Thus, we will not describe Fig. 4 in as much detail.

The opposite orientation of the Burgers vectors of the two MDs is a necessary but not sufficient condition for their annihilation. To annihilate, two MDs must become close enough for the interaction of DW distortions in their cores to cause their magnetostatics-mediated mutual attraction.

The frames presented in Fig. 5 (1–4) illustrate the case of an interaction between two MDs with their similarly oriented Burgers vectors $\overrightarrow{b}$. A comparison of frames 1 and 3 in Fig. 5 reveals that MD II overtakes MD I. As a consequence of the similar orientations of $\overrightarrow{b}{\rm{s}}$, the annihilation is not possible.

### Quantitative parameters of magnetic dislocations

Observations show that MDs are dynamic formations. We introduce a series of quantitative parameters to describe them (see the Methods section).

**Figure 6:**
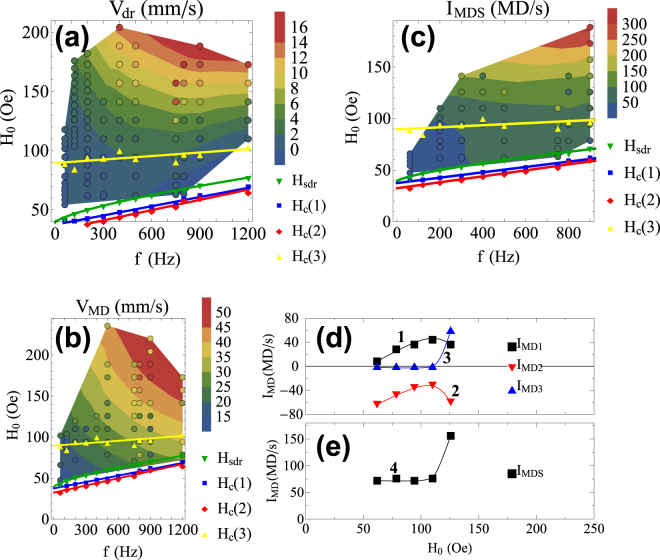
Dependencies of magnetic dislocation quantitative parameters on the pump field frequency and amplitude. (**a**–**c**) Amplitude-frequency diagrams of the speed of the drift of stripe domains *V*_*dr*_, the average speed of magnetic dislocations *V*_*MD*_ and total activity of centers of nucleation of magnetic dislocations *I*_*MDS*_, correspondingly. The surfaces in the diagrams are second-order polynomial fits of experimental data points that are shown with colored markers (filled circles). The superimposed graphs are frequency dependencies of amplitudes at which the drift begins *H*_*sdr*_ and corresponding nucleation centers are activated *H*_*c*_. (**d**,**e**) Dependencies on pump field amplitude (*f* = 500 Hz): (**d**)–activities of centers of nucleation of magnetic dislocations (*I*_*MD*1−3_), (**e**)–net (total) activity of centers of nucleation of magnetic dislocations No. 1–3. *I*_*MD*1_ rises and reaches its maximum value of 44 MDs/s at *H*_0_ = 110 Oe (curve 1). At the same time, the modulus of the activity of center No. 2 decreases and reaches its minimum value of 32 MDs/s at *H*_0_ = 110 Oe (curve 2).

The amplitude-frequency dependencies of quantitative parameters *V*_*dr*_, *V*_*MD*_ and *I*_*MDS*_ are shown in the diagrams of Fig. 6(a–c). For the activation fields of centers of nucleation 1, 2, and 3 and the drift-starting amplitude *H*_*sdr*_, the following relation was preserved throughout our research: *H*_*c*_(1) ≈ *H*_*c*_(2) < *H*_*sdr*_ < *H*_*c*_(3), i.e., centers No. 1 and 2 are activated virtually simultaneously; at higher amplitudes, the drift of DWs begins; and at *H*_*c*_(3), center No. 3 is activated.

*V*_*dr*_ shown in Fig. 6(a) increases vs *H*_0_. A particularly strong increase is observed at *H*_0_ > *H*_*c*_(3). *V*_*MD*_ shown in Fig. 6(b) exhibits a virtually linear increase versus both amplitude and frequency. Note that the movement speeds of MDs are several times greater than drift speeds (by a factor of 4.2 for maximum values of *V*_*MD*_ and *V*_*dr*_). *I*_*MDS*_ shown in Fig. 6(c) remains virtually constant between *H*_*c*_(2) and *H*_*c*_(3) and begins to increase at *H*_*c*_(3) simultaneously with an increase of *V*_*dr*_.

Consider the activities of nucleation centers in more detail. The direction of the movement of MDs from centers No. 1 and 3 is the same as the drift direction of DWs (see Fig. 1), and the movement direction of MDs from center No. 2 is opposite of the drift direction.

For the frequency *f* = 500 Hz, the activities *I*_*MD*_ of MD nucleation centers are measured, and the results are shown in Fig. 6(d,e). Centers No. 1 and 2 compensate each other up to a field amplitude of *H*_0_ = 110 Oe (curves 1 and 2 in Fig. 6(d)). At *H*_0_ = 110 Oe, center No. 3 is activated (curve 3 in Fig. 6(d)). At *H*_0_ > 110 Oe, the total number of MDs nucleated in the sample during one second sharply increases (curve 4 in Fig. 6(e)).

Note that the speed of the drift of DWs considerably increases, starting from approximately *H*_0_ = 110 Oe (Fig. 6(a)). For this frequency, the value of the drift speed of DWs saturates upon further increasing *H*_0_. This result could be attributed to a balance between domains that formed and moved out of the crystal.

## Discussion

### Mechanism of movement of magnetic dislocations based on vertical Bloch lines

**Figure 7:**
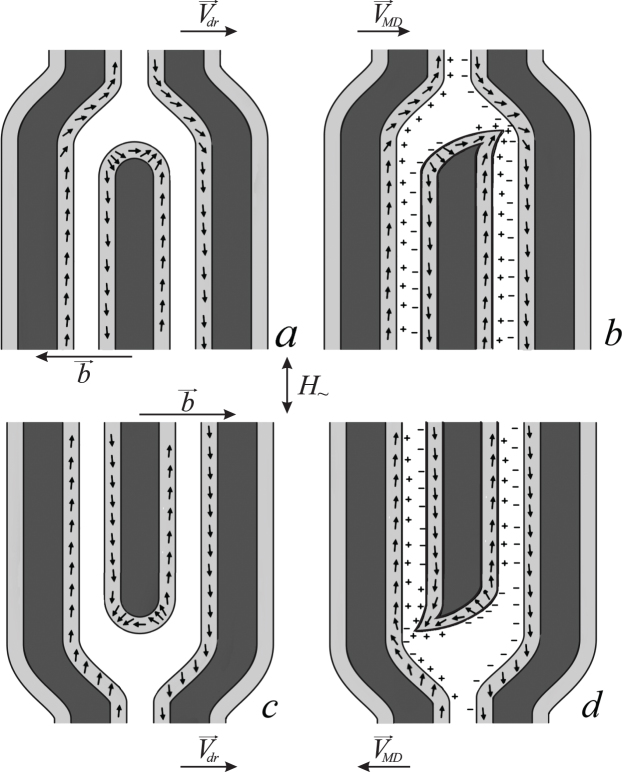
Distribution of magnetization vectors in domain walls near a magnetic dislocation. (**a**,**b**) A magnetic dislocation moving to the right. (**c**,**d**) A magnetic dislocation moving to the left.

To explain the observed transversal movement of MDs, we propose a mechanism based on an MD core structure model that includes vertical Bloch lines (VBLs) (Fig. 7). Examples of possible magnetization distributions around an MD for the case when the drift is present and directed to the right are shown in Fig. 7(a) and (c). In this model, we assume that the magnetic structure of the sample is two-dimensional based on a study^19^ that concluded that the domain structure in samples of iron garnets of this thickness is homogeneous throughout thickness and open. We take the results of magnetic force microscopy studies of the structure of static DWs that we have performed into account. Those results indicate that neighboring walls have opposite polarity, i.e., their average magnetizations lie in the plane of the plate and are directed along the two opposite directions.

We assume the existence of a pair of VBLs in the apexes of MDs. A VBL is a transition area between two segments of a Bloch domain wall in which the magnetization rotates clockwise and counterclockwise. A model of such a DW structure is shown in Fig. 3.87 in^2^ and in Fig. 2S in the Supplementary Materials. Note that the formation of vertical Bloch lines in a domain wall is a well-known and common phenomenon. This term is widely used in the scientific literature (see^20,21^, for example).

The most important aspect in the proposed mechanism for the movement of MDs is a certain spatial distortion of an MD core cross-section, i.e., elongation of a short stripe domain toward a neighboring stripe domain.

It is known^22^ that a DW requires an average magnetization component *M*_*n*_ normal to the DW plane to appear in order to move. In accordance with this requirement, magnetization in most DWs during drift will be inclined toward their movement direction. *M*_*n*_ creates a magnetostatic field, and the magnetization moves around it in a precession. This mechanism for the movement of DWs is valid for both constant and alternating fields^23^. During the stationary movement of a DW (the speed of a DW does not depend on time), the *M*_*n*_ component will not change with time. The drift is a stationary movement of all DWs with a constant speed and in the same direction. Therefore, the average magnetization of a DW will be inclined toward the drift direction. This is valid for one-dimensional, two-dimensional and three-dimensional domain walls^24,25^.

Due to the presence of the two VBLs in the apex of a shortened domain, the apex will not only shift to the right but will also deform as shown in Fig. 7(b). Interaction between magnetostatic charges leads to merging of a shortened domain and the right dark domain. Thus, a new, bright MD is formed. Exactly this type of MD deformation and the formation of a new MD is observed experimentally when an MD moves along the drift of DWs (scheme in Fig. 2).

The movement of MDs in the direction opposite to the drift is observed experimentally (from nucleation center No. 2). To explain this observation, we assume that the number density of poles is higher on an MD near a VBL than on the curved parts of neighboring stripe domain walls. In this case, the apex of a shortened stripe domain will be distorted in the way shown in Fig. 7(c,d) and reorient the magnetization on the right side of the left curved stripe domain by its field. Therefore, this dark stripe domain and the shortened domain will merge and form a bright MD, i.e., the MD will shift to the left.

The necessity of breaks in periodically repeated domain walls is a feature of the transversal movement of MDs that results in the switching of MD core magnetization in the direction opposite to the initial one and in a shift of the MD to a distance equal to a width of one domain. In contrast, the longitudinal movement of MDs (along DWs) is realized through a continuous shift of the MD core (see Supplementary Movie 2S); this creates a DS distortion that moves through a sample together with the core. During this process, the length of a shortened domain is changing, and no breaks occur in domain walls. When an MD reaches the edge of a sample, DS distortions disappear, and the structure becomes regular. Note that in Supplementary Movie 2S, an MD moves along DWs with a speed of *V* = 19.13 mm/s and across DWs with a speed of *V* = 7.20 mm/s. The drift speed of domain walls is *V*_*dr*_ = 0.05 mm/s.

### Frequency dependencies of quantitative parameters of magnetic dislocations

The nucleation of MDs begins only when some frequency-dependent critical field amplitude *H*_*c*_ is reached. The higher the frequency is, the higher is the critical field amplitude at which the nucleation of MDs begins (see Fig. 6(a)).

The obtained frequency dependencies of the activation field amplitudes for the centers of nucleation of MDs (Fig. 6(a)) can be explained as follows.

When domain walls move to some distance far enough from each other, the excess magnetostatic field begins to affect this domain. In some areas of the crystal (e.g., with lower anisotropy energy), this can stimulate the formation of an area with reversed magnetization. The higher the $\overrightarrow{H}$ is in this location of the crystal, the more probable is the nucleation process. However, the nucleation of a local area of reversed magnetization is associated with an $\overrightarrow{M}$ rotation process (inhomogeneous in this case), which can proceed only with a limited speed. Correspondingly, increasing *H*_0_ increases the nucleation probability, but the event itself may not occur if the field is changing fast enough. Nucleation can be made possible at a given field change speed if its amplitude is increased.

According to our observations, the magnetic dislocation nucleation process proceeds as follows: a nucleus of an inverse phase is formed, it grows and becomes a shortened domain, and we describe its movement as the motion of an MD. The inverse phase formation process was repeatedly discussed^26^. It was theoretically shown that an inverse phase nucleates in areas of a crystal where anisotropy or exchange energy is lower. Such areas may be, for example, located near structural defects.

## Conclusion

This paper is the first to experimentally consider the dynamic behavior of a stripe domain structure with active centers of nucleation of magnetic dislocations subjected to an AC magnetic field at frequencies of 25–1200 Hz. The following have been discovered:

1. Magnetic dislocations are able to move not only along magnetic domains but also perpendicular to domain walls. The movement of MDs along and across domain walls is realized with different mechanisms but is observed simultaneously. When an MD moves across domain walls, its magnetization orientation switches interchangeably.
2. Magnetic dislocations interact with one another. The processes of the annihilation, mutual passing of magnetic dislocations through each other, and their overtaking one another are revealed. The interaction of MDs occurs when they approach each other to distances on the order of the sizes of the cores of magnetic dislocations.
3. Magnetic dislocations are formed in different nucleation centers at field amplitudes *H*_0_ > *H*_*c*_, where *H*_*c*_ is the critical field amplitude at which the nucleation of MDs in centers begins. It depends linearly on the external field frequency and is defined by the characteristics of the nucleation centers.
4. The amplitude of the alternating magnetic field applied along a stripe structure at which all stripe domains start moving perpendicularly to domain walls is higher than the amplitude at which the nucleation of MDs begins for all studied frequencies. This result indicates a stimulation of the drift of DWs by the process of nucleation of MDs.

The obtained experimental data on the activity of MD nucleation centers indicate that it depends on the AC magnetic field amplitude and frequency. Field amplitudes at which the centers of nucleation of MDs are activated and at which the drift of DWs begins increase as the frequency is increased. Moreover, the value of the drift speed of DWs significantly increases in the AC field amplitude region where the total activity of the centers of nucleation of MDs sharply increases. This result again indicates an important role of MDs in stimulating the drift of DWs.

The proposed VBL mechanism explains the discovered features of the behavior of magnetic dislocations: MD movement across DWs, symmetry break in the selection of MD movement direction, and interaction of MDs.

A new mechanism for the change in magnetization distribution during the dynamic magnetization and remagnetization of a crystal is proposed. It is the movement of an MD in a domain structure accompanied by switching of the magnetization of an MD.

The discovered features of the behavior of MDs as topological defects have an undoubted effect on the dynamics of domain structures. Meanwhile, the features of the dynamic behavior of MDs can be generalized for other magnetic inhomogeneities.

## Methods

### Samples

The dynamic behavior of magnetic dislocations was studied at room temperature on iron garnet single-crystal (111) and (110) plates of different compositions. Single crystals of iron garnets were grown from solutions of melts following the synthesis technique described in^27^. The results presented in this paper were obtained on a 73 μm thick plate that was cut from a (TbErGd)_3_(FeAl)_5_O_12_ single crystal such that the crystallographic [111] axis was perpendicular to the surface of the plate-like sample. A Faraday rotation for a 655-nm wavelength was measured using a photometric technique^28^ (linearly polarized light passes the sample at normal incidence and then passes the analyzer installed at *π*/4 with respect to the polarizer) and equals 0.05 deg/μm.

The samples had natural cubic crystallographic anisotropy with the constant *K*_1_ and four easy magnetization axes (EMAs) oriented along four diagonals of a cubic cell. One of the easy magnetization axes was located along the [111] axis, and the other EMAs were made with the surface of a crystal the same angles. Moreover, the samples had growth-induced rhombic anisotropy with one EMA coinciding with the [111] axis; the other EMA is $[\bar{2}\mathrm{11]}$–projection of $[\bar{1}\mathrm{11]}$ to the surface of the crystal. This anisotropy was characterized by two constants: *K*_*u*_ and *K*_*p*_. Numerical values of all constants that characterize the sample studied are presented in Table 1, as well as those of the saturation magnetization *M*_*S*_ and the quality factor ($Q={K}_{u}\mathrm{/2}\pi {M}_{S}^{2}$).

**Table 1:** Magnetic and geometrical characteristics of (111) plate (TbErGd)_3_(FeAl)_5_O_12_.

Thickness *L*, *μm*	*M*_*S*_, GS	*K*_*u*_, erg/cm^3^	*K*_1_, erg/cm^3^	*K*_*p*_, erg/cm^3^	Q	*K*_u_/*K*_1_	*K*_u_/*K*_p_
73	40	6500	4400	3200	0.55	1.5	2.0

The equilibrium DS of this sample is represented–in a freeze-frame video–by a regular system of bright- and dark-lighted images of stripe domains elongated along the $[\bar{2}\mathrm{11]}$ axis. The “bright” and “dark” domains were characteristic of the resulting magnetization vectors oriented in the direction of the normal $\overrightarrow{n}$ to the top sample surface and in the opposite direction (−$\overrightarrow{n}$), respectively. Consequently, magnetic poles of positive (negative) sense form on the top surfaces of bright (dark) domains (see scheme in Supplementary Fig. 2S).

### Experimental setup and procedures

A sample with a stripe DS was exposed to an alternating (in time *t*) magnetic field ${H}_{\sim }={H}_{0}\,\sin \,\mathrm{(2}\pi ft)$ applied in the sample plane along stripe domains (along their DWs). The linear frequency *f* of this field was varied in the range 25–1200 Hz, and the amplitude *H*_0_ reached 250 Oe. A dynamic domain structure was observed with a magneto-optical Faraday effect and recorded using a high-speed camera (at rates up to 1000 fps). A 655-nm diode 100 mW laser was employed for visualizing the dynamic domain structure. Values of the velocity of the drift of domain walls (*V*_*dr*_), the velocity of movement of magnetic dislocations (their averaged value *V*_*MD*_), and the activity of centers of nucleation of MDs (*I*_*MD*_) were measured by digital processing of experimental videos using algorithms specifically designed for this application.

### Measured quantities

To provide a quantitative description of the observed processes, we have introduced the following parameters:

- **MD velocity**;
- **Velocity of the drift** of domain walls ${\overrightarrow{{V}}}_{dr}$;
- **Individual and predominant movement trajectories** of the MDs nucleated in specific centers;
- The MD **Burgers vector** $\overrightarrow{b}$;
- The **average movement speed** *V*_*MD*_ of MDs. We calculate *V*_*MD*_ as the average movement speed of all MDs observed at a given alternating magnetic field amplitude *H*_0_. Averaging was performed over all MDs detected during one second in the observed sample area (Fig. 1), and their maximal number was 316 MDs/s (equal to *I*_*MDS*_ in Fig. 6(c)). On average, the number of measurements for each MD was about eight;
- The **activity** *I*_*MD*_ of MD nucleation centers that is defined as the number of MDs nucleated in this center during one second. At low field amplitudes, the nucleation centers of MDs are inactive (*I*_*MD*_ = 0).

The activity of centers No. 1 and 3 (*I*_*MD*1_ and *I*_*MD*3_) from which MDs move in the same direction as DWs during drift (see Fig. 1) is considered positive, and the activity *I*_*MD*2_ of center No. 2 from which MDs move in the direction opposite to the drift of DWs is negative. The total activity of MD nucleation centers in a sample can be characterized by the sum of modules of the activities *I*_*MDS*_ of centers No. 1, 2, and 3.

Measurements of *V*_*dr*_, *V*_*MD*_, and *I*_*MDS*_ shown in Fig. 6(a–c) were performed with digital processing of one-second length movies; measurements of *H*_*sdr*_ and the fields of activation of the centers of nucleation were performed with direct observation on a timescale of minutes. Due to this difference, the values of threshold amplitudes appear to have been underestimated compared to the *V*_*dr*_, *V*_*MD*_, and *I*_*MDS*_ dependencies: observations on a timescale of minutes allow detecting the nucleation of MDs even when *I*_*MDS*_ is far below 1 MDs/s, which is the lowest value detectable by software in our experiment. This is why the curves *H*_*c*_(2) and *H*_*c*_(1) in Fig. 6(a–c) lie below regions where *V*_*dr*_, *V*_*MD*_, and *I*_*MDS*_ were measured.

### Interpretation of domain structures

A magnetic dislocation that is discussed in the current paper is most easily imagined if one considers a system of straight mutually parallel stripe domains with one of them shorter than the others (“analog” of an atomic half-plane inserted between other planes).

The MD Burgers vector $\overrightarrow{b}$ is introduced as in^12^, i.e., it can be found following a geometrical rule. If one follows a closed contour in a clockwise direction on a domain structure consisting of regular vertical stripes and counts crossed periods of a domain structure as steps, then a similar contour consisting of the same steps but with an MD inside will not be closed due to the additional shortened domain that enters this contour from one side. The additional step required to close the contour is the MD Burgers vector $\overrightarrow{b}$. Its length is equal to the domain structure period. $\overrightarrow{b}$ characterizes an individual MD and is not positioned in a domain structure, although it is aligned with respect to it. Its direction is perpendicular to straight DWs and, in a domain structure mostly consisting of vertical stripes, is pointing to the left for an MD with its shortened domain pointing up (see the inset in Fig. 1). In this case, the direction of $\overrightarrow{b}$ reverses if the entire domain structure is mirrored vertically.

### Representation of experimental data

The experimental image sets of a dynamic DS presented in this paper do not show the full sets of all stages of movement of MDs because of hardware limitations of high-speed video recording. The frames shown in different panels within Figs 2, 3, 4 and 5 are displayed without any lateral shifts between panels such that the drift of DWs is visible (frames in panels were taken from the same location of a sample).

### Data availability statement

The datasets generated during and/or analyzed during the current study are available from the corresponding author on reasonable request.

## Electronic supplementary material

Movie 1S

Movie 2S

Movie 3S

Supplementary materials
