# Supplementary figures and images for: Motion and Interaction of Magnetic Dislocations in Alternating Magnetic Field
Source: Sci Rep. 2017 Dec 22;7:18084. doi: 10.1038/s41598-017-18033-2 (PMC5741713; doi:10.1038/s41598-017-18033-2)

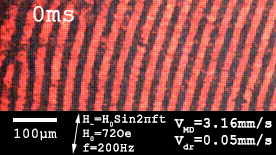

Supplement: Supplementary file 1 — Movie 1S [file 41598_2017_18033_MOESM1_ESM.gif]

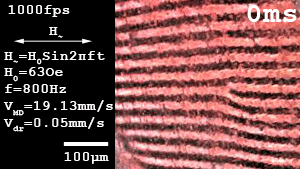

Supplement: Supplementary file 2 — Movie 2S [file 41598_2017_18033_MOESM2_ESM.gif]

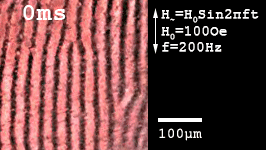

Supplement: Supplementary file 3 — Movie 3S [file 41598_2017_18033_MOESM3_ESM.gif]
