# Supplementary material for: Motion and Interaction of Magnetic Dislocations in Alternating Magnetic Field
Source: Sci Rep. 2017 Dec 22;7:18084. doi: 10.1038/s41598-017-18033-2 (PMC5741713; doi:10.1038/s41598-017-18033-2)
Supplement: Supplementary file 4 — Supplementary materials [file 41598_2017_18033_MOESM4_ESM.pdf]

# Supplementary materials: Motion and Interaction of Magnetic Dislocations in Alternating Magnetic Field

**L. A. Pamyatnykh[1,*], B. N. Filippov[2,1], L. Y. Agafonov[1], and M. S. Lysov[1]**

[1]Ural Federal University, Lenin Av. 51, Ekaterinburg, 620083, Russia
[2]Mikheev Institute of Metal Physics, Ural Branch, Russian Academy of Sciences, Ekaterinburg, 620219, Russia
[*]Lidia.Pamyatnykh@urfu.ru

## ABSTRACT

The behavior of magnetic dislocations (MDs) in an alternating harmonic magnetic field in iron garnets has been experimentally investigated. The results are presented for single-crystal plates in which the drift of domain walls is observed in fields of sound frequencies.
It is found that MDs in a stripe domain structure are able to move not only along but also across domain walls. A pairwise interaction between magnetic dislocations when they approach each other to distances on the order of the sizes of the cores of MDs is revealed. The processes of the annihilation, mutual passing of magnetic dislocations through each other and overtaking are found. The features of the dynamic behavior of MDs are explained using a mechanism based on the presence of vertical Bloch lines in a structure of domain walls. MDs are formed at nucleation centers, and their nucleation field is lower than the drift-starting field, which corresponds to previously proposed dislocational mechanism of the drift.
The dependencies of quantitative parameters of the drift and MDs on amplitude and frequency of the pumping field are determined. The behavior of MDs should be considered when analyzing the mechanisms for magnetization and temperature-dependent phase transitions in magnetic layers.

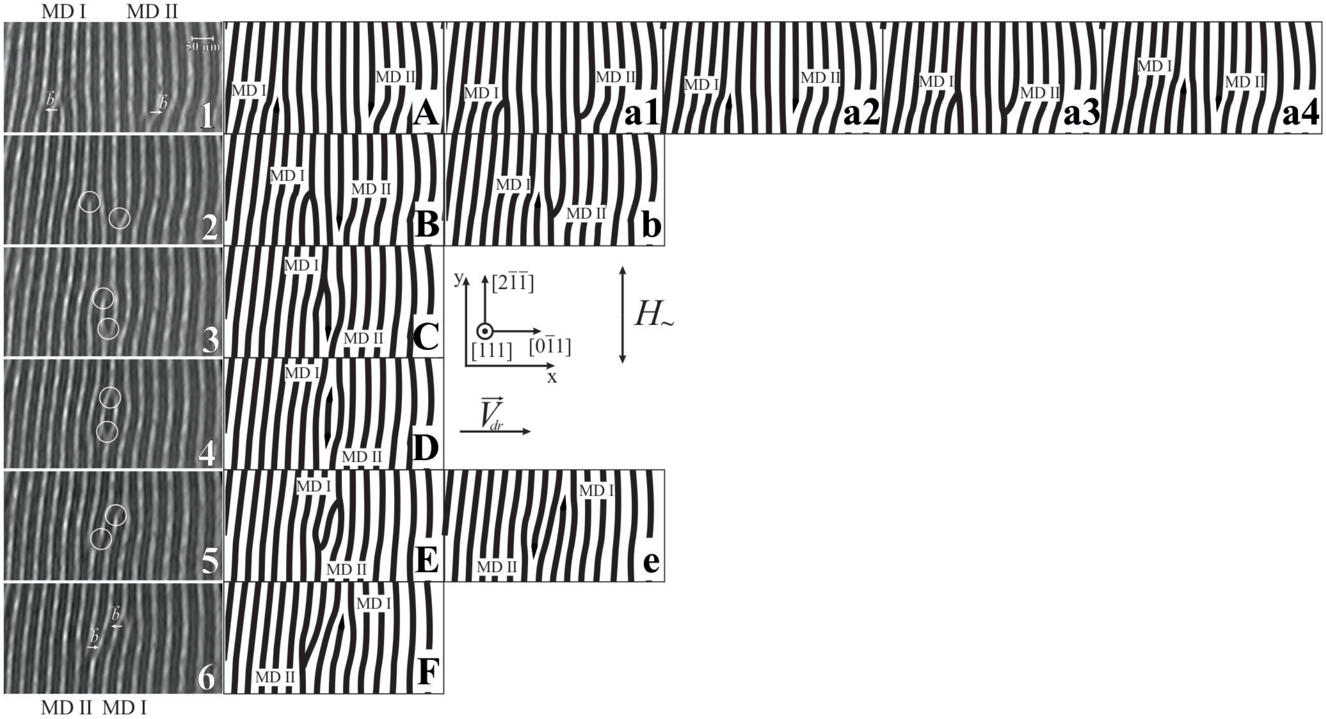


**Figure 1S. Two magnetic dislocations moving toward each other.** The Burgers vectors $\vec{b}$ of magnetic dislocations are aligned in opposite directions. **1**-**6**, Experimental magneto-optical images of an iron garnet sample domain structure in an AC magnetic field. The AC field frequency is $f = 60$ Hz, and the amplitude is $H_0 = 106$ Oe. The Burgers vectors of magnetic dislocations are shown in (**1**, **6**). The cores of magnetic dislocations are encircled in **2**-**5**. The time between frames equals (**1**-**2**) 40, (**2**-**3**) 20, (**3**-**4**, **4**-**5**, and **5**-**6**) 10 ms. **A**-**F**, Schematic representation of corresponding domain structure states. **a1**-**a4**, **b**, **e**, Supposed intermediate states between frames (**1**, **2**), (**2**, **3**) and (**5**, **6**) based on observations, correspondingly.

**Movie 1S. Transversal movement of a single magnetic dislocation in a system of stripe domains.** The movement is exerted by an external AC magnetic field $H_\sim = H_0 \sin(2\pi f t)$ with $H_0 = 72$ Oe and $f = 200$ Hz. The movie is composed of frames that were obtained in the same AC field phase near $H_\sim = 0$. The speed of the drift of stripe domains $V_{dr} = 0.05$ mm/s; the velocity component of the magnetic dislocation along the drift direction $V_{MD} = 3.16$ mm/s; the velocity component of the magnetic dislocation along the domain wall orientation $V_{MD} = 0.44$ mm/s.

**Movie 2S. Longitudinal movement of a single magnetic dislocation in a system of stripe domains.** The movement is exerted by an external AC magnetic field $H_\sim = H_0 \sin(2\pi f t)$ with $H_0 = 63$ Oe and $f = 800$ Hz. The speed of the drift of stripe domains $V_{dr} = 0.05$ mm/s; the velocity component of the magnetic dislocation along the drift direction $V_{MD} = 19.13$ mm/s; the velocity component of the magnetic dislocation along the domain wall orientation $V_{MD} = 7.20$ mm/s.

**Movie 3S. Interaction between two magnetic dislocations moving toward each other in a system of stripe domains.** The movement is exerted by an external AC magnetic field $H_\sim = H_0 \sin(2\pi f t)$ with $H_0 = 100$ Oe and $f = 200$ Hz. The cores of magnetic dislocations are encircled during slower replay.

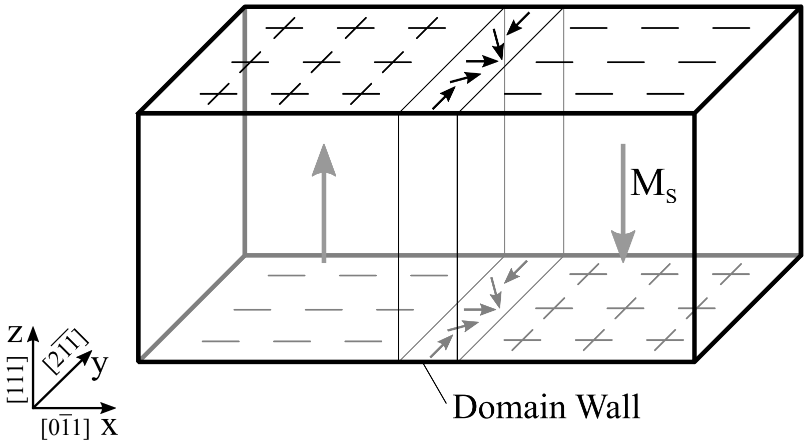


**Figure 2S.** **A scheme of magnetization distribution near a vertical Bloch line.** The sample is a single-crystal plate that was cut from a $(TbErGd)_3(FeAl)_5O_{12}$ crystal such that the crystallographic [111] axis is perpendicular to the surface. The sample has a stripe domain structure with domain walls oriented along the $[2\bar{1}\bar{1}]$ axis, which is the projection of the easy magnetization axis $[1\bar{1}\bar{1}]$ onto the plane of the sample. Here, the [111] axis is denoted as $Z$ and is referred to as ”vertical”. The $Y$ axis is along $[2\bar{1}\bar{1}]$; thus, domain walls are in plane $YZ$. The $X$ axis is perpendicular to the planes of domain walls and coincides with the crystallographic direction $[0\bar{1}1]$. The magnetization vector is directed parallel or antiparallel to $Z$ in domains and rotates in plane $YZ$ inside Bloch domain walls. This rotation may be either clockwise or anticlockwise. When one Bloch domain wall has segments of both types, they are separated with vertical Bloch lines where the magnetization has a nonzero $X$ component. The magnetization is assumed to be independent of the $Z$ coordinate.
